# Synergy between Vancomycin and Nafcillin against *Staphylococcus aureus* in an In Vitro Pharmacokinetic/Pharmacodynamic Model

**DOI:** 10.1371/journal.pone.0042103

**Published:** 2012-07-24

**Authors:** Steven N. Leonard

**Affiliations:** 1 School of Pharmacy, Bouvé College of Health Sciences, Northeastern University, Boston, Massachusetts, United States of America; 2 Department of Pharmacy, Brigham and Women’s Hospital, Boston, Massachusetts, United States of America; Harvard Medical School, United States of America

## Abstract

**Introduction:**

Continued pressure from glycopeptide use has led to non-susceptible strains of *Staphylococcus aureus* including heterogeneously vancomycin-intermediate *S. aureus* (hVISA). Infections with hVISA are associated with poor patient outcomes, thus incentivizing novel treatments. Evidence suggests that vancomycin and anti-staphylococcal penicillin susceptibility are inversely related which indicates that the use of this combination may be particularly useful against methicillin-resistant *S. aureus* with reduced susceptibility to vancomycin, such as hVISA. The aim of this study was to evaluate the potential for synergy between vancomycin and nafcillin against hVISA.

**Methods:**

Twenty-five hVISA strains were evaluated for vancomycin and nafcillin minimum inhibitory concentration (MIC) by broth microdilution in duplicate. Potential for synergy was assessed by time-kill at 1/2x MIC in triplicate. Five strains were chosen, representing the range nafcillin MIC’s available in the cohort –4, 16, 64, 128, and 256 µg/mL, and were run in an *in vitro* pharmacokinetic/pharmacodynamic (PK/PD) model in duplicate over 72 hours to evaluate the potential of the combination with simulated human pharmacokinetics. In addition, 4 fully glycopeptide susceptible strains of *S. aureus* including 2 methicillin-susceptible (MSSA) and 2 methicillin-resistant (MRSA) were run in the PK/PD model for comparison.

**Results:**

In the time-kill, 92% of strains (23 of 25) displayed synergy with the combination of vancomycin and nafcillin. In the PK/PD model, all five strains of hVISA showed an improvement in overall activity (P≤0.004) and organism burden at 72 hours (P≤0.001) with the combination compared to either drug alone. The combination was also successful against both MRSA and MSSA in overall activity (P≤0.009) and organism burden at 72 hours (P≤0.016), though the magnitude of the effect was diminished against MSSA.

**Conclusions:**

The combination of vancomycin and nafcillin significantly improved antibacterial activity against hVISA, MRSA, and MSSA compared to either drug alone.

## Introduction

The worldwide dissemination and poor treatment outcomes of methicillin-resistant *Staphylococcus aureus* (MRSA) presents therapeutic difficulties for clinicians. Historically vancomycin has been the mainstay of therapy for MRSA infections, however decades of selective pressure has led to evolutionary changes in *S. aureus* diminishing the utility of this agent. [1], [2], [3], [4], [5], [6], [7] Of note is the emergence of heterogeneously vancomycin intermediate *S. aureus* (hVISA); a particularly concerning organism as it is not detected by traditional susceptibility testing or automated systems commonly utilized in clinical microbiology laboratories. [4], [8], [9] Due to these detection difficulties the true prevalence is difficult to estimate but generally ranges from 5–15% (although this varies widely based on geographic location, testing method used, time period of isolates tested, etc.). [4], [9], [10], [11] It has also been shown that the prevalence of hVISA may be rising. [9] This is concerning as preliminary studies have found an association between infection with hVISA and poor treatment outcomes including prolonged fever and bacteremia, increased length of hospital stay, vancomycin treatment failure, and longer total duration of antibiotic therapy. [1], [2], [3], [4], [5], [6], [11].

The use of combination antimicrobial therapy is a common occurrence and represents a potential treatment option for infections caused by hVISA. [10] Multiple guidelines from the Infectious Diseases Society of America (IDSA) advocate for the use of a myriad of combination antimicrobial therapies for different purposes. [12], [13], [14], [15] The clinical use of combination therapy for MRSA, outside of the clinical practice guidelines above, has become ubiquitous and thus there is an ongoing need to characterize antimicrobial interactions to find the most potentially useful combinations. Several previous investigations have found synergy between beta lactams and anti-MRSA agents including vancomycin, daptomycin, and telavancin against MRSA. [16], [17], [18], [19], [20], [21] These combinations have been explored because, clinically, the use of an antistaphylococcal penicillin is desirable in the setting where beta lactams have activity. [22], [23] There are also reports showing an inverse relationship between vancomycin and beta lactam susceptibility, indicating that the use of beta lactam combinations may be particularly useful against organisms with reduced susceptibility to vancomycin, such as hVISA. [24], [25], [26] The objective of this investigation was to evaluate the potential for synergy between vancomycin and nafcillin against hVISA by time kill analysis and further evaluate the combination with an in vitro pharmacokinetic/pharmacodynamic (PK/PD) model utilizing realistic drug concentrations and pharmacokinetics.

**Figure 1:**
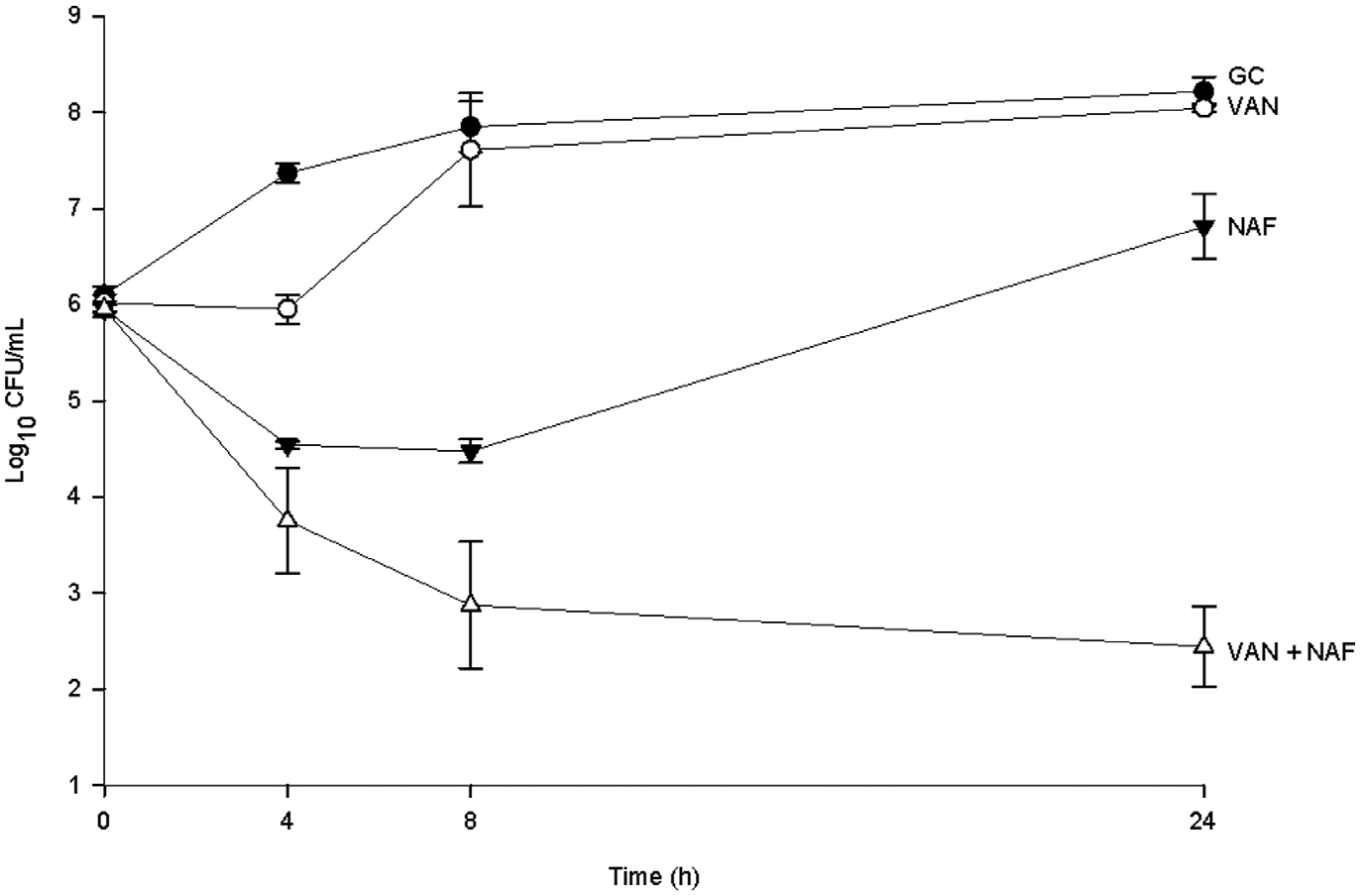
Time–kill curve analysis of one hVISA (R3003, VAN MIC = 2, NAF MIC = 256 µg/mL) isolate. All antimicrobials are at a concentration of 0.5x MIC. This isolate shows synergy between vancomycin and nafcillin. Data are presented as a graph of the mean bacteria remaining vs. time with error bars at the sampling points representing 1 standard deviation from the mean. GC = Growth Control, NAF = Nafcillin, VAN = Vancomycin.

## Materials and Methods

### Bacterial Strains

Twenty five clinical isolates of hVISA already proven positive by population analysis area under the curve ratio using Mu3 as a positive control (PAP-AUC) (provided by the Anti-Infective Research Laboratory, Detroit, MI) were utilized for susceptibility and time kill experiments. [9] Isolates were collected between 1986 and 2007 from several hospitals throughout metropolitan Detroit (Detroit Receiving Hospital and Henry Ford Hospital, Detroit, MI, and William Beaumont Hospital, Royal Oak, MI) and from the SENTRY antimicrobial surveillance program. Five strains were selected from the above described cohort of 25 strains and run in a one compartment *in vitro* pharmacokinetic/pharmacodynamic (PK/PD) model. In addition, 4 fully glycopeptide susceptible clinical isolates of *S. aureus* including 2 methicillin-susceptible (MSSA) and 2 methicillin-resistant (MRSA) were run in the PK/PD model for comparison. These 4 isolates were collected from Brigham and Women’s Hospital, Boston, MA in 2010.

### Antimicrobial Agents

Vancomycin and nafcillin were purchased from a commercial source (Sigma Chemical Company, St. Louis, MO).

### Media

Mueller-Hinton broth (Difco, Detroit, MI) supplemented with 25 mg/L of calcium, 12.5 mg/L magnesium, and 2% sodium chloride (due to the presence of nafcillin and according to CLSI recommendations) (SMHB) was used for all susceptibility testing, time kills, and PK/PD models. [27] Colony counts were determined using Tryptic Soy Agar (TSA, Difco, Detroit, MI). Mueller Hinton Agar (MHA, Difco, Detroit, MI) was used to test for the emergence of resistance.

**Figure 2:**
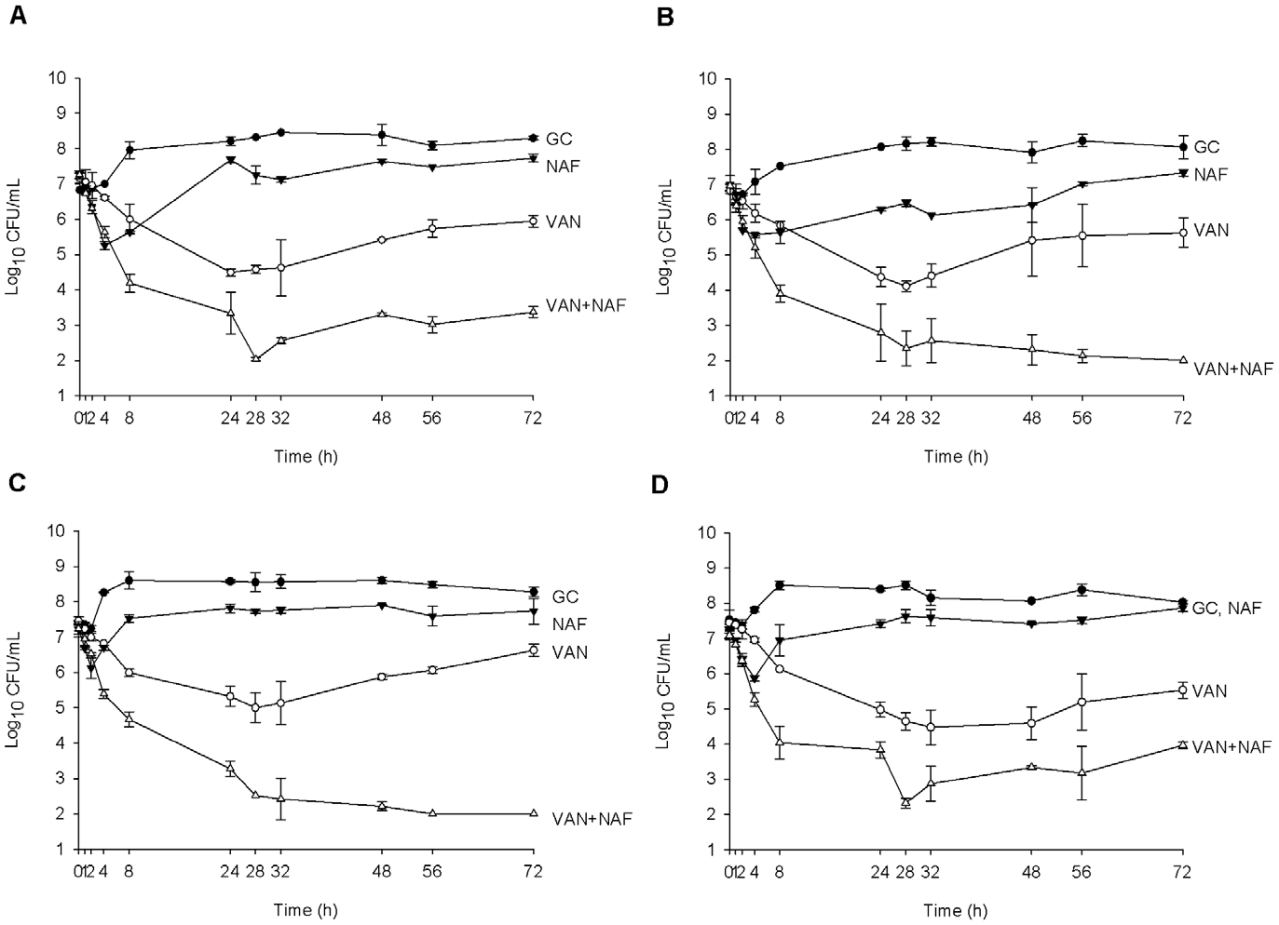
Activity of vancomycin and nafcillin alone and in combination in the PK/PD model against hVISA strains where the concentration of nafcillin was below the MIC of the respective organism for 100% of the dosing interval. Graphs shown are of isolate R5253 (Nafcillin MIC = 16 µg/mL) (A), R1915 (Nafcillin MIC = 64 µg/mL) (B), R2729 (Nafcillin MIC = 128 µg/mL) (C), and R3003 (Nafcillin MIC = 256 µg/mL) (D). Data are presented as a graph of the mean bacteria remaining vs. time with error bars at the sampling points representing 1 standard deviation from the mean. GC = Growth Control, NAF = Nafcillin, VAN = Vancomycin.

### Susceptibility Testing

Minimum inhibitory concentrations (MIC) of study antimicrobial agents were determined by broth microdilution at ∼5.5 log_10_ CFU/mL according to Clinical and Laboratory Standards Institute (CLSI) guidelines. [27].

### Synergy Testing

Potential for synergy with vancomycin plus nafcillin was determined by time-kill methods in triplicate at a final inoculum of ∼10^6^ CFU/mL. All time-kill experiments were performed at 1/2x the MIC of the respective antibiotic. Aliquots (0.1 ml) were removed at 0, 4, 8, and 24 hours, serially diluted in 0.9% sodium chloride, and plated on TSA plates with a lower limit of detection of 2 log_10_ CFU/mL. Time-kill curves were constructed by plotting mean colony counts (log_10_ CFU/ml) versus time. Synergy was defined as ≥ 2-log_10_ CFU/mL increase in killing at 24 hours with the combination, in comparison with the killing by the most active single drug. Combinations that resulted in ≥1-log_10_ bacterial growth in comparison to the least active single agent were considered to represent antagonism. All combinations not meeting the definition of synergy or antagonism were considered indifferent. All samples were incubated at 37°C for 24 hours.

### In Vitro Pharmacokinetic/Pharmacodynamic (PK/PD) Infection Model

Five strains of hVISA, one from each available nafcillin MIC in the cohort –4 (isolate R1629), 16 (R5253), 64 (R1915), 128 (R2729), and 256 (R3003) µg/mL, were chosen to be run in an *in vitro* PK/PD model consisting of a 125 mL one-compartment glass apparatus with ports for the addition and removal of media, antibiotics, and samples. These strains were selected to represent the full continuum of nafcillin susceptibility available in order to determine if there was a beta lactam susceptibility “ceiling” to any enhanced killing effect observed. In addition, two strains of methicillin-susceptible *S. aureus* (MSSA; isolates SNL4 and SNL9) and two strains of methicillin-resistant *S. aureus* (MRSA; isolates SNL96 and SNL98), all fully glycopeptide susceptible, were run in the PK/PD model as comparators. The model was placed in a water bath at 37°C throughout the simulation with a magnetic stir bar for mixing. Fresh media was continuously supplied and removed via a peristaltic pump (Masterflex, Cole-Parmer Instrument Company, Chicago, IL) set to simulate the half-lives of the antibiotics. A starting inoculum of ∼10^7^ CFU/mL was used for all simulations. This higher inoculum was chosen because hVISA requires a high inoculum to observe the heterogeneous phenotype and to provide a more rigorous experimental condition for vancomycin and nafcillin, both of which are subject to an inoculum effect on their activity. [28], [29] Free drug concentrations were used to simulate regimens of vancomycin 1 g every 12 h (targets: *f*C_max_: 30 µg/mL, *f*C_min_: 7.5 µg/mL, half-life: 6 h; at 50% protein binding for vancomycin these levels correspond to a total C_max_ of 60 µg/mL and C_min_ of 15 µg/mL) [30], nafcillin 2 g every 4 h (targets: *f*C_max_: 5.2 µg/mL, *f*C_min_: 0.325 µg/mL, half-life: 1 h; at 87% protein binding these levels correspond to a total C_max_ of 40 µg/mL and C_min_ of 2.5 µg/mL) [31], [32], and vancomycin 1 g every 12 h combined with nafcillin 2 g every 4 h. The vancomycin dose was chosen to simulate a total drug trough of 15–20 µg/mL to conform to the recent vancomycin dosing guidelines stating target trough values should fall within this range for most infections. [30] The nafcillin dose is the standard dose used to treat serious staphylococcal infections. Model simulations involving two drugs with different half-lives were performed using a previously validated method. [33] All models were done in duplicate to ensure reproducibility.

**Figure 3:**
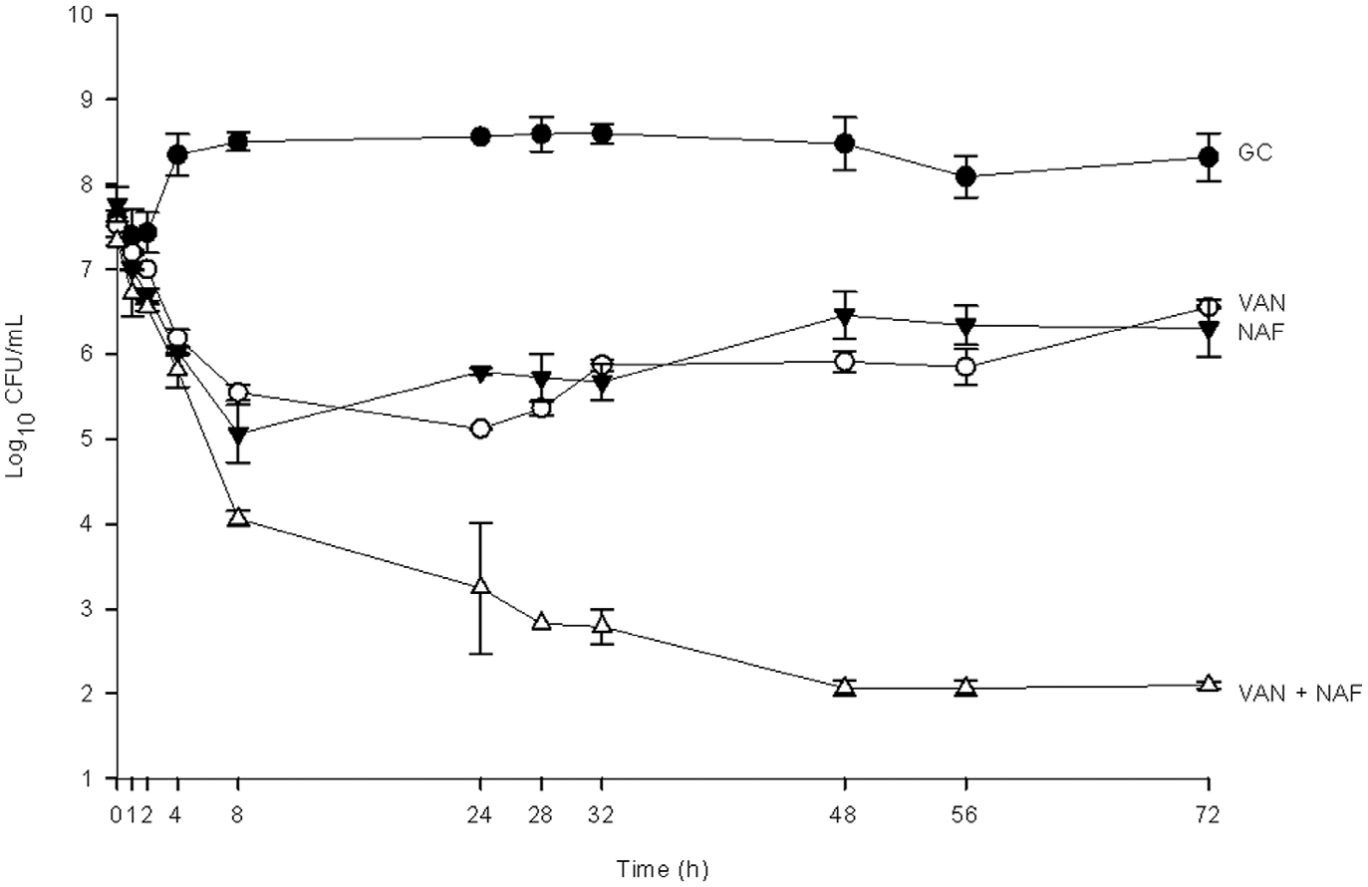
Activity of vancomycin and nafcillin alone and in combination in the PK/PD model against the hVISA strain with a nafcillin MIC of 4 µg/mL (R1629). Data are presented as a graph of the mean bacteria remaining vs. time with error bars at the sampling points representing 1 standard deviation from the mean. GC = Growth Control, NAF = Nafcillin, VAN = Vancomycin.

### Pharmacodynamic Analysis

Samples (approximately 1 mL each) were drawn from each model at 0, 1, 2, 4, 8, 24, 28, 32, 48, 56, and 72 h, serially diluted in 0.9% sodium chloride, and plated on TSA plates for quantification with a lower limit of detection of 2 log_10_ CFU/mL. Antibiotic carryover was accounted for using serial dilutions. The total reduction in log_10_ CFU/mL was determined by plotting time-kill curves of the number of remaining organisms over the 72 hour time period. Bactericidal activity was defined as ≥ 3 log_10_ CFU/mL (99.9%) reduction in colony count from initial inoculum. The time to achieve a 99.9% bacterial load reduction (T_99.9_) was determined by linear regression (r^2^≥0.95) or by visual inspection.

### Pharmacokinetic Analysis

Pharmacokinetic samples were obtained, through the injection port over 72 h for verification of target antibiotic concentrations. Concentrations of vancomycin were measured by bioassay utilizing *Bacillus subtilis* ATCC 6633. [34] Nafcillin concentrations were measured by bioassay utilizing *Micrococcus luteus* ATCC 9341 as previously described. [29] The elimination half-lives (t_1/2_), areas under the curve (AUC), peaks (*f*Cmax), and troughs (*f*Cmin) were determined using WinNonlin PK/PD modeling software program (Pharsight, Cary, NC, USA).

**Figure 4:**
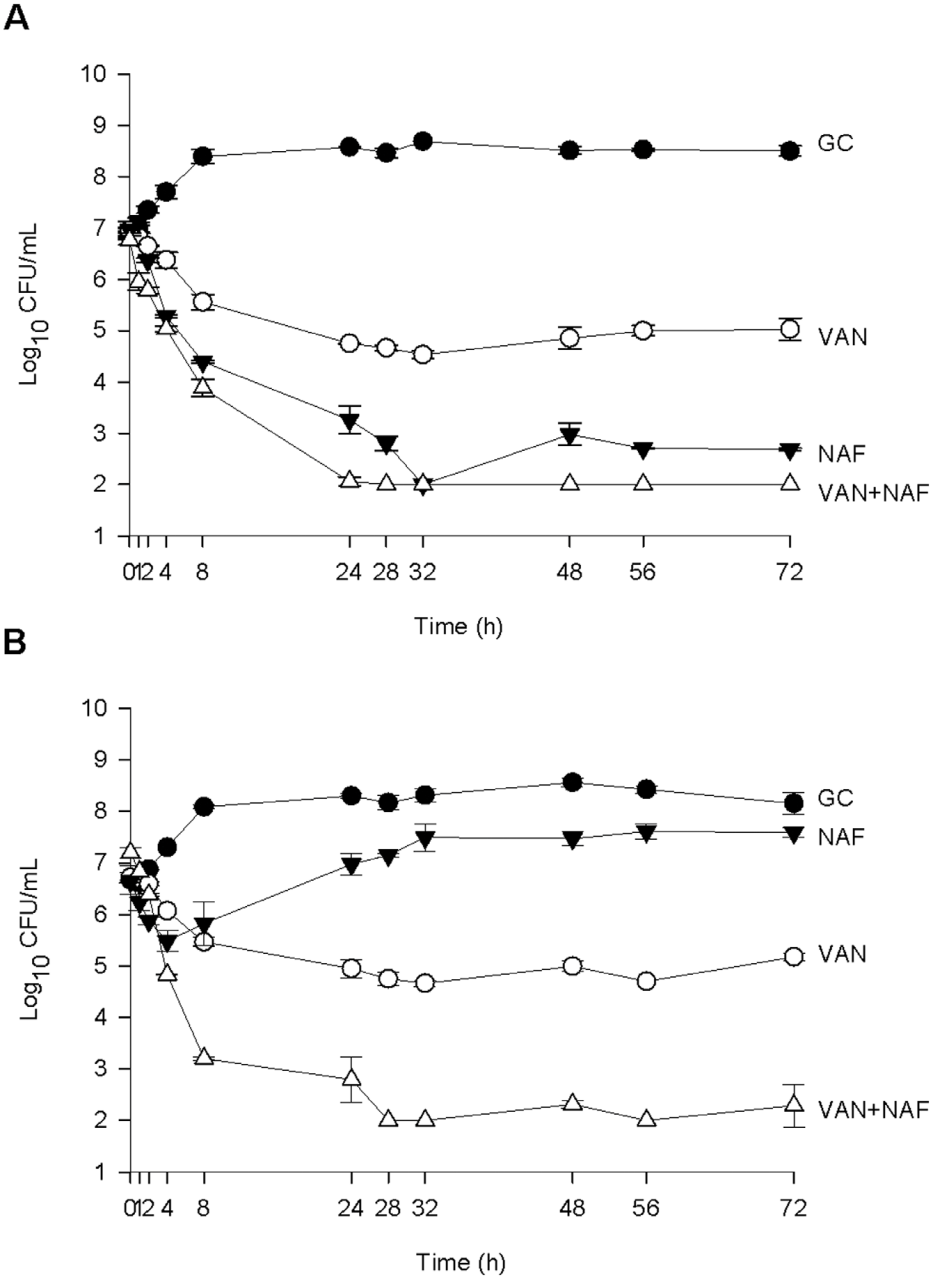
Activity of vancomycin and nafcillin alone and in combination in the PK/PD model against one MSSA isolate (SNL9) (A) and one MRSA isolate (SNL96) (B). Data are presented as a graph of the mean bacteria remaining vs. time with error bars at the sampling points representing 1 standard deviation from the mean. GC = Growth Control, NAF = Nafcillin, VAN = Vancomycin.

### Resistance

Development of resistance was evaluated at multiple time points throughout the simulation at 24, 48, and 72 hours. One hundred µL samples from each time point were plated on Mueller Hinton agar plates containing 3 fold the MIC of the respective antibiotic to assess the development of resistance. Plates were then examined for growth after 48 hours of incubation at 35°C. The MIC for observed growth was measured by broth microdilution. In addition, growth from quantification plates at 24, 48, and 72 h was subjected to MIC testing by broth microdilution.

### Statistical Analysis

Overall activity of regimens over the 72 hour period was compared by calculating the area under the killing curve (AUC) for each regimen using SigmaPlot software (version 11.1, Systat Software Inc., San Jose, CA). The AUCs were then compared using analysis of variance (ANOVA) with Tukey’s post-hoc test. Additionally, changes in log_10_ CFU/mL at the 72 hour time point were compared using ANOVA with Tukey’s post-hoc test. All statistical comparisons were done with IBM SPSS Statistics (Version 19.0, SPSS Inc., Chicago, IL). A P value of ≤0.05 was considered significant.

**Table 1:** Area under the bacterial kill curve values from the PK/PD models.

Strain	GrowthControl	Vancomycin	Nafcillin	Vancomycin +Nafcillin
R1629	603.8±3.1	420.4±2.7	431.2±4.8	216.1±8.6
R5253	585.3±4.8	391.8±11.5	512.5±0.4	248.5±7.8
R1915	570.4±8.3	377.6±14.6	462.0±1.7	205.6±14.5
R2729	609.6±6.4	422.5±0.6	549.2±4.5	216.8±8.5
R3003	592.1±1.3	378.8±15.0	530.1±6.0	262.3±12.4
SNL96	590.8±0.5	365.5±2.4	507.7±8.8	192.6±4.9
SNL98	568.8±1.7	372.9±2.8	515.5±2.3	226.3±11.0
SNL4	610.6±1.3	384.0±4.3	288.4±7.8	218.3±11.9
SNL9	607.0±2.0	364.6±0.6	233.3±1.5	184.5±1.9

Results are presented as mean ± standard deviation.

## Results

For the 25 isolates of hVISA the vancomycin MIC_50_ (MIC_50_ = median MIC) and MIC_90_ (MIC_90_ = MIC at which 90% of strains were inhibited) were both 2 µg/mL (range 1–2 µg/mL) and the nafcillin MIC_50_ and MIC_90_ were 128 and 256 µg/mL respectively (range 4–256 µg/mL). In time kill analysis the addition of nafcillin to vancomycin showed synergy in 92% of strains (23/25) with the remaining strains showing indifference. An example time kill graph is displayed in figure 1.

All 5 hVISA strains selected for the PK/PD model had a vancomycin MIC of 2 µg/mL, and, as described above, were selected to represent the full range of nafcillin MIC available in the cohort of 25 strains. All 5 strains displayed synergy in time kill analysis. Both MRSA isolates used in the PK/PD model had a vancomycin MIC of 1 µg/mL while one (MRSA SNL96) had a nafcillin MIC of 32 µg/mL and the other (MRSA SNL98) had a nafcillin MIC of 128 µg/mL. Likewise, both MSSA isolates used had a vancomycin MIC of 1 µg/mL and both had a nafcillin MIC of 0.5 µg/mL. Pharmacokinetic analysis demonstrated the accuracy of the models performed with PK parameters within 10% of targeted values. The free peak (*f*C_max_), trough (*f*C_min_), and half-life of vancomycin obtained in the PK/PD model were (all data presented as mean ± standard deviation throughout) 32.8±3.2 µg/mL, 8.2±2.3 µg/mL (corresponding with a total drug trough mean of 16.4 µg/mL), and 6.2±0.5 h respectively. The free peak (*f*C_max_) and half-life of nafcillin were 4.8±0.7 µg/mL and 1.1±0.2 h.

In the PK/PD model against the 5 hVISA strains (figures 2 and 3); vancomycin alone demonstrated similar activity between isolates resulting in maximal killing of between 2–3 log_10_ CFU/mL from baseline between 24 and 32 hours and thereafter showing regrowth. Likewise, nafcillin alone displayed generally similar activity against 4 of the 5 isolates displaying 1–2 log_10_ CFU/mL kill in the first 4 hours, followed by regrowth (figure 2). Against the isolate with the lowest nafcillin MIC (MIC = 4 µg/mL; figure 3) nafcillin displayed similar activity to vancomycin alone (P = 0.54) resulting in maximal kill at 8 hours followed by slow regrowth over the remaining 64 hours of the experiment. Several changes in MIC were noted with vancomycin alone and with nafcillin alone. For 3 of the 5 strains (those with baseline nafcillin MICs of 16, 64, and 128 µg/mL) the vancomycin MIC changed from 2 µg/mL to 4 µg/mL by 72 hours. Three of the 5 strains also showed a one dilution step increase in nafcillin MIC by 72 hours (strains with an original nafcillin MIC of 4, 64, and 128 µg/mL changed to 8, 128, and 256 µg/mL respectively). Although all of these MIC changes were confirmed through replicates of testing as described above, it is generally considered that one dilution step is within the standard margin of error for an MIC test and therefore these results should be interpreted with caution. Against all 5 isolates, the combination of vancomycin and nafcillin was superior to either drug alone overall (P≤0.004 for all comparisons) and at the 72 hour time point (P≤0.001 for all comparisons). The time to bactericidal activity for the combination was 7.7±0.4 h while no individual drug achieved bactericidal activity alone. No changes in MIC were observed for the combination of vancomycin and nafcillin for either drug or any of the 5 strains over the 72 hour period. Two of 5 strains (R1915 and R2729) were killed to detection limits (2 log_10_ CFU/mL) at 72 hours.

Against the MRSA and MSSA strains (Figure 4), the activity of vancomycin alone was similar across all 4 strains, and indeed was similar to the activity of vancomycin alone against the hVISA strains. The difference, however, was that no changes in MIC were observed for any of the MRSA and MSSA over the 72 hours for either vancomycin or for nafcillin with any experimental regimen. As expected, the activity of nafcillin alone against MRSA was minimal while the activity of nafcillin against MSSA was significantly better than that of vancomycin alone overall (P≤0.003) and at the 72 hour time point (P≤0.001). The combination of vancomycin and nafcillin was significantly better than either vancomycin or nafcillin alone against both MRSA (P≤0.001 for overall activity, P≤0.005 at the 72 hour time point) and both MSSA (P≤0.009 for overall activity, P≤0.016 at the 72 hour time point). For MRSA the time to bactericidal activity for the combination was 6.3±0.5 h while no individual drug achieved bactericidal activity alone. Against MSSA the time to bactericidal activity for the combination was 10.2±2.2 h and was 14.3±0.8 h for nafcillin alone. Vancomycin alone was not bactericidal against MSSA. Kill to detection limits at 72 hours was achieved for one MSSA strain (SNL9) and neither MRSA strain. All AUC values from PK/PD model experiments are displayed in table 1.

## Discussion

*Staphylococcus aureus* remains a common cause of a variety of infections causing high morbidity and mortality. [35] Throughout much of the world, including such places as the United States, several European countries, much of South America, Australia, and Japan, the prevalence of methicillin resistance in *S. aureus* is quite high. [36] Due to the high prevalence in these areas, beta lactams cannot be used empirically and therefore vancomycin is generally considered the standard of care for suspected staphylococcal infections. This level of vancomycin selective pressure has led to an increasing problem with decreased susceptibility to vancomycin in *S. aureus*, including hVISA.

Consistent with previous investigations, we found vancomycin to be mostly ineffective against hVISA strains, failing to produce bactericidal activity and resulting in MIC elevations in several strains. [28], [37], [38] However, when vancomycin was combined with nafcillin, strong enhancement of bacterial killing was observed for all 5 strains examined. This is in spite of nafcillin concentrations being below the MIC of the organism for most (strain with nafcillin MIC 4 µg/mL) to all (all remaining strains) of the dosing interval. This is a peculiar because the time drug concentrations are above the MIC of the organism is what drives antibacterial activity for beta lactams. [39] One potential reason for this observation is that the hindrance of peptidoglycan synthesis, in this case by vancomycin, can reduce beta lactam resistance. [24] Though this is the case, the exact mechanism of synergy between beta lactams and glycopeptides has not been precisely elucidated to date.

Likewise, against MRSA and MSSA we observed this same enhancement in killing with the combination of vancomycin and nafcillin. Against all of these strains, similar to hVISA, vancomycin alone failed to produce bactericidal activity while nafcillin was not effective at all against MRSA and was quite effective alone against MSSA, as expected. This strong activity of nafcillin against MSSA accounts for the fact that, while the combination of vancomycin and nafcillin was better than either drug alone, the magnitude of the difference was much less for MSSA than it was for both MRSA and hVISA strains that were not susceptible to beta lactams.

The observation of synergy between beta lactams and vancomycin is not new, though the combinations have only been described once previously using simulated human pharmacokinetics. [21] In that study vancomycin was combined with cefazolin against 2 MRSA strains, one hVISA strain, and one vancomycin intermediate *S. aureus* strain and the combination of the 2 drugs was found to improve overall activity, but not bacterial density at the end of the experiments (48 and 72 hours). One potential reason for this disparity is that the two studies used different vancomycin dosing regimens. In their investigation, they used every 8 hour dosing as opposed to every 12 hours, however, given that the trough values in both studies were similar and that massive increases in AUC/MIC ratio have been shown not to result in improvements in vancomycin activity, [28] this seems unlikely to be the reason. Another difference was that they used a different beta lactam agent (cefazolin vs. nafcillin) which may have been less active than nafcillin against *S. aureus*. A final possibility is that the starting inoculums differed between the two studies, with a higher starting inoculum in the present investigation. Given that both glycopeptides and beta lactams are subject to an inoculum effect, [28], [29] where the killing effect of an antibiotic is lessened as the inoculum of organism increases, this could have led to the diminished activity of vancomycin in this study compared to their investigation where vancomycin displayed much more kill alone.

In conclusion, the combination of vancomycin and nafcillin significantly improved overall antibacterial activity, rate of bacterial killing, and remaining organism burden at 72 hours against MSSA, MRSA, and hVISA isolates over either drug alone. This improvement was seen even when the isolate was very resistant to nafcillin (susceptible breakpoint for *S. aureus* is MIC ≤2 µg/mL) and the nafcillin time above MIC was zero. These data support the continued evaluation of this combination, and its potential role in the treatment of *S. aureus* infections.
